# A familial case report of 17q12 recurrent deletion syndrome: clinical and molecular characterization

**DOI:** 10.3389/fendo.2026.1819047

**Published:** 2026-05-12

**Authors:** Yaroslav V. Dvoryanchikov, Rita I. Khusainova, Ildar R. Minniakhmetov, Ramil R. Salakhov, Kirill V. Smirnov, Saida A. Ibragimova, Ivan I. Golodnikov, Elena A. Sechko, Ekaterina A. Dobreva, Natalia G. Mokrysheva

**Affiliations:** Endocrinology Research Centre, Moscow, Russia

**Keywords:** 17q12 recurrent deletion syndrome, CNV, diabetes mellitus, HNF1B, MODY 5

## Abstract

This article presents the first reported familial case of 17q12 recurrent deletion syndrome in Russia, involving a female patient with diabetes and her daughter diagnosed with atypical autism without intellectual disability. A comprehensive analysis of the molecular genetic features and intrafamilial variability of clinical manifestations was performed. In addition, clinical, laboratory, and instrumental findings were compared with those observed in a classical case of maturity-onset diabetes of the young type 5 (MODY5). Furthermore, the disease course associated with other genetic alterations affecting the 17q12 region, including 17q12 microduplications and various pathogenic variants of the *HNF1B* gene, was comparatively evaluated. Given the orphan nature of this condition, the present report adds to the limited existing data on the clinical and genetic characteristics of 17q12 recurrent deletion syndrome.

## Introduction

17q12 Deletion Syndrome (OMIM: 614527), also referred to as 17q12 recurrent deletion syndrome or 17q12 microdeletion syndrome (1), is characterized by variable combinations of three core features: structural or functional abnormalities of the kidneys and urinary tract, maturity-onset diabetes of the young type 5 (MODY5), and neurodevelopmental or neuropsychiatric disorders, including intellectual disability or developmental delay, autism spectrum disorder (ASD), attention deficit hyperactivity disorder, and other psychiatric manifestations. The estimated prevalence of the syndrome is approximately 1:50,000 in the European population and 1:25,000 in the United States (2).

The 17q12 region is considered a hotspot for copy number variants (CNVs), defined as deletions or duplications of contiguous genomic regions that may cause genomic disorders, including Mendelian diseases, or be associated with complex multifactorial conditions (3). The size of the deletion ranges from 1.3 to 1.8 Mb and may result in the loss of multiple genes, including *AATF, ACACA, C17ORF78, DDX52, DHRS11, DUSP14, GGNBP2, HNF1B, LHX1, MRM1, MYO19, PIGW, SYNRG, TADA2A*, and *ZNHIT3* (4). This genetic variability contributes to heterogeneity in the clinical manifestations of the syndrome.

Multicystic kidney dysplasia and other structural or functional renal abnormalities are observed in 85-90% of affected individuals, MODY5 in approximately 40%, and developmental delay or learning difficulties in about 50% (5). Neuropsychiatric manifestations, including ASD, schizophrenia, behavioral disturbances (aggression and self-injury), and seizures, have also been reported, with seizures occurring in up to 36% of patients. Additional features include microcephaly, visual abnormalities, endocrine disorders, short stature, and renal and cardiac anomalies (6).

Loss-of-function variants in the *HNF1B* gene lead to renal cysts and diabetes syndrome, also known as MODY5, which is typically diagnosed before the age of 25 years (range 10–50 years). The first pathogenic *HNF1B* variant (R177X) was described in a Japanese family in 1997, and in 2001 the condition was designated renal cysts and diabetes (RCAD) syndrome (7). RCAD syndrome (OMIM #137920) demonstrates a broad clinical spectrum due to the multisystem role of *HNF1B* in the development of the kidneys, pancreas, liver, bile ducts, and urogenital tract (8). RCAD accounts for less than 5% of all MODY cases, and its clinical heterogeneity frequently results in misdiagnosis as type 1 or type 2 diabetes (9).

To our knowledge, this is the first reported familial case of 17q12 recurrent deletion syndrome in Russia, identified in a female patient with diabetes and her daughter. Given the orphan nature of this condition, the description of individual cases contributes to a more comprehensive characterization of the disease, expanding the limited existing data on clinical variability and genetic heterogeneity associated with 17q12 deletion syndrome.

## Materials and methods

Genomic DNA was extracted from peripheral blood leukocytes using the MagPure Blood DNA Kit (Magen, China). DNA concentration and purity were assessed using a Nano500 spectrophotometer (Alsheng, China). Initially, targeted next-generation sequencing (NGS) was performed. Sequencing was carried out on the Illumina NovaSeq 6000 platform (Illumina, San Diego, CA, USA) using paired-end sequencing (2 × 150 bp). The targeted NGS panel covered the coding regions of canonical transcripts of selected genes, as well as 25 nucleotides of the adjacent intronic regions flanking each exon. The panel included the following genes: *ABCC8, AKT2, ALMS1, ARMC5, BLK, CACNA1D, DIS3L2, EIF2AK3, FOXA2, GATA6, GCG, GCGR, GCK, GLIS3, GLUD1, GPC3, HADH, HNF1A, HNF1B, HNF4A, IGF1, IGF1R, INS, INSR, KCNJ11, KDM6A, KLF11, LIPE, MC3R, MC4R, NEUROD1, NSD1, PAX4, PDX1, PGM1, PIK3CA, PPARG, PPP1R3A, PTF1A, RFX6, SH2B1, SIM1, SLC16A1, TUB, UCP2, WFS1*, and *ZFP57*. Library preparation was performed using the KAPA HyperPlus Kit (Roche, Basel, Switzerland) according to the manufacturer’s instructions. Variant classification was performed in accordance with the guidelines of the American College of Medical Genetics and Genomics (ACMG) (10).

To validate the identified genomic deletion, chromosomal microarray analysis (CMA) was performed. Sample processing followed the protocol provided with the CytoScan™ Optima Array reagent kit (Thermo Fisher Scientific, Waltham, MA, USA). Arrays were scanned using the GeneChip System 3000 system for nucleic acid analysis (Thermo Fisher Scientific, United States). Primary data analysis was conducted using Chromosome Analysis Suite (ChAS) software, version 4.5 (Thermo Fisher Scientific, Waltham, MA, USA).

## Results

During genetic testing for hereditary forms of diabetes using a targeted NGS gene panel, a large heterozygous deletion on chromosome 17 was identified in the proband. The deletion was initially detected with approximate boundaries at chr17:36937923-37745159 (hg19), spanning 807,236 bp and fully encompassing the *ACACA* and *HNF1B* genes. At the time of genetic testing, the patient had an initial clinical diagnosis of type 1 diabetes (T1DM).

**Figure 1:**
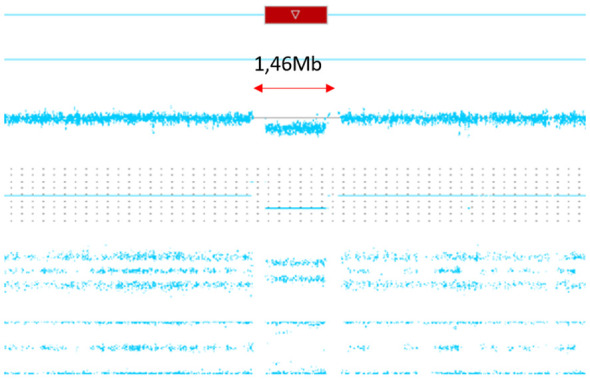
Graphical representation of the chromosomal microarray analysis results detected deletion with approximate boundaries at chr17:36937923-37745159 (hg19), spanning 807,236 bp and fully encompassing the ACACA and HNF1B genes for the index patient’s daughter.

To confirm and further characterize this finding, CMA was performed in the proband as well as in her two daughters and her mother (the father was unavailable for testing). CMA revealed a 17q12 microdeletion of 1,466,767 bp (chr17:36388435-37855202; hg19) in the proband and her elder daughter, while no deletion was detected in the other tested family members (**Figure 1**).

## Case presentation

At the age of 26, during a routine health examination, the patient was found to have an elevated glycated hemoglobin (HbA1c) level of 7.1%. An initial diagnosis of T1DM was considered; however, autoantibody testing was not performed at that time. In the absence of marked insulin deficiency, dietary therapy with dynamic monitoring of carbohydrate metabolism was recommended. Under dietary management, target glycemic control was achieved, with HbA1c levels ranging from 5.7% to 6.7%.

At the age of 29, the patient became pregnant and underwent further evaluation. Testing for antibodies to glutamic acid decarboxylase (GADA) and insulin (IAA) yielded negative results. During pregnancy, glycemic control remained within target ranges on dietary therapy, with HbA1c levels of 5.3-5.5%. However, at 29–30 weeks of gestation, emergency delivery was required due to fetal hypoxia. The neonate exhibited unstable hemodynamics and marked glycemic fluctuations, which were associated with progressive clinical deterioration and subsequent death. Autopsy revealed multiple congenital anomalies, including pancreatic hypoplasia with nesidioblastosis, meconium ileus, and multicystic dysplasia of both kidneys. CMA performed in the child did not identify pathogenic microdeletions or microduplications larger than 400 kb, nor regions of loss of heterozygosity involving imprinted genes.

Three years later, the patient became pregnant again. During the first and second trimesters, carbohydrate metabolism remained within target ranges under dietary therapy. In the third trimester, episodes of fasting hyperglycemia developed, necessitating initiation of long-acting insulin therapy. Prenatal ultrasound at 36 weeks of gestation revealed bilateral polycystic kidney changes in the fetus, with 1–2 mm cysts in both renal pelvises without pelvic dilatation.

At the age of 36 (2021), the patient experienced her third pregnancy. Long-acting insulin therapy was required during the first and second trimesters, and short-acting insulin was added in the third trimester. A healthy female infant was delivered, with no congenital anomalies detected. The child’s growth and neurodevelopment were appropriate for age.

In 2023, the patient underwent targeted NGS of a gene panel for hereditary forms of diabetes. The analysis identified a heterozygous deletion on chromosome 17 with approximate boundaries at chr17:36937923-37745159 (hg19), spanning 807,236 bp and fully encompassing the *ACACA* and *HNF1B* genes. To confirm this finding using a confirmatory method, CMA was performed and detected a 17q12 microdeletion extending from chr17:36,388,435 to 37,855,202 (hg19), with a total size of 1,466,767 bp.

To assess inheritance, CMA was additionally performed in the patient’s mother and both daughters (the father was unavailable for testing). The analysis revealed the same microdeletion in the patient’s elder daughter, while no pathogenic copy number variants were detected in the patient’s mother or younger daughter.

Based on the CMA findings and the progressive deterioration of carbohydrate metabolism, long-acting insulin therapy was initiated. A comprehensive inpatient evaluation was subsequently performed. Longitudinal data on carbohydrate metabolism parameters from 2011 to 2024 are presented in **Supplementary 1**.

## Clinical and laboratory characteristics of the index patient

At the time of evaluation, the patient was 39 years old. Parameters of carbohydrate metabolism exceeded the individual target levels: HbA1c was 8.7% (individual target <6.5%), while the C-peptide concentration was 1.31 ng/mL, indicating preserved endogenous insulin secretion. Glucose-lowering therapy was adjusted, and gliclazide at a dose of 30 mg once daily in the morning was prescribed, resulting in improved glycemic control and good tolerability (**Table 1**).

**Table 1:** Glycemic values (mmol/L) during therapy with gliclazide 30 mg and insulin glargine 300 U/mL (8 U administered in the evening).

Morning		Afternoon		Evening		
08:00	10:30	13:00	15:30	17:00	19:30	21:30
–	8,2	6,3	12,3	9,5	11,8	9,9
8,4	7,2	8,3	8,4	6,9	9,0	8,2
6,9	8.7	7,8	4,9	4,7	8,6	8,9
5,7	9,5	6,2	6,7	7,3	6,5	9,7

Screening for chronic complications of diabetes was performed. Renal filtration function was mildly reduced: serum creatinine was 79.5 µmol/L, and the estimated glomerular filtration rate (eGFR), calculated using the CKD-EPI equation, was 80 mL/min/1.73 m². Analysis of a spot urine sample showed an albumin-to-creatinine ratio of 0.4 mg/mmol (reference range 0–3.5), with no evidence of diabetic nephropathy. Renal ultrasound revealed sonographic signs of multiple renal parenchymal cysts. No signs of diabetic retinopathy were detected. Distal symmetric sensory polyneuropathy was diagnosed. Duplex ultrasound of the brachiocephalic arteries demonstrated non-stenotic atherosclerotic changes in the extracranial segments.

Given previously reported data on secondary hyperparathyroidism in patients with 17q12 microdeletion syndrome, calcium-phosphate metabolism was assessed. Albumin-corrected serum calcium was at the lower limit of normal (2.17 mmol/L; reference range 2.15-2.55), phosphorus was 1.19 mmol/L (0.74-1.52), and parathyroid hormone level was 41.5 pg/mL (15-65), indicating no evidence of hyperparathyroidism. Blood biochemistry revealed hypomagnesemia (0.49 mmol/L; reference range 0.70-1.05) and low-normal potassium levels (3.77 mmol/L; reference range 3.50-5.10). Therapy with potassium aspartate and magnesium aspartate (158 mg + 140 mg, one tablet three times daily) was initiated.

Given reports suggesting that hypertriglyceridemia may represent a metabolic abnormality in patients with 17q12 deletion syndrome, a comprehensive lipid profile was evaluated. Total cholesterol was 5.3 mmol/L, low-density lipoprotein cholesterol 3.34 mmol/L, high-density lipoprotein cholesterol 1.56 mmol/L, and triglycerides 0.9 mmol/L. A moderate elevation of aspartate aminotransferase (AST) was observed (48.4 U/L; reference range 5.0-34.0). Abdominal ultrasound demonstrated diffuse structural changes of the pancreas. No clinical or instrumental evidence of neuropsychiatric disorders was identified.The results of laboratory and instrumental examinations are presented in **Supplementary 2**.

Overall, the clinical and laboratory characteristics of the index patient are consistent with previously reported features of 17q12 deletion syndrome. A notable aspect of the present case is the availability of detailed longitudinal monitoring of carbohydrate metabolism from the onset of diabetes, demonstrating that sustained glycemic control within target ranges can be achieved with dietary therapy for an extended period following disease manifestation.

## Clinical and laboratory characteristics of the index patient’s daughter carrying a 17q12 deletion

A nephrologist has followed the elder daughter since early childhood due to prenatally detected renal abnormalities. Renal ultrasound performed at the age of 4 years revealed multiple small cystic lesions in both kidneys, consistent with multiple renal cysts. Longitudinal laboratory data obtained prior to hospitalization are presented in **Supplementary 3**.

At the age of 7 years, the patient was hospitalized for comprehensive evaluation. Renal hyperfiltration was identified, with a serum creatinine level of 38.7 µmol/L and an estimated glomerular filtration rate of 161.8 mL/min/1.73 m², calculated using the Schwartz formula. Renal ultrasound demonstrated multiple parenchymal renal cysts and pyelectasis of the left kidney. A moderate elevation of AST was observed (49.7 U/L; reference range 13-35), while alanine aminotransferase (ALT) levels were at the upper limit of normal (33.1 U/L; reference range 7-35). No abnormalities of carbohydrate metabolism were detected; C-peptide concentration was within the low-normal range for age (1.08 ng/mL; reference range 1.0-4.8). Mild myopia was diagnosed. Assessment of calcium-phosphate metabolism revealed persistently elevated alkaline phosphatase levels for age, reaching 433.4 U/L (reference range 160-319). The serum magnesium level was 0.73 mmol/l (reference range 0.7 – 1.05), ionized calcium 1.3 mmol/L (reference range 1.05-1.3), phosphorus 1.6 mmol/L (reference range 1.29-2.26), uric acid 229.8 µmol/l (reference range 154.7-357). The complete set of laboratory and instrumental findings is provided in **Supplementary 4**.

Neuropsychological evaluation performed by a medical psychologist identified moderate impairments in executive functioning, including difficulties with planning, regulation, and control of goal-directed activity, reduced inhibitory control, and challenges in task switching. Additional findings included moderate deficits in kinesthetic processing and mild impairments in auditory information processing, while these secondary features were less pronounced. Psychiatric assessment, conducted according to ICD-10 criteria, resulted in a diagnosis of F84.1 (atypical autism without intellectual disability) accompanied by expressive language impairment. Pharmacological therapy with risperidone was initiated at a dose of 0.25 mg twice daily.

Confirmation of the chromosomal abnormality by CMA enabled expansion of the patient’s follow-up program to include regular assessment of carbohydrate metabolism, in addition to the previously established nephrological monitoring. This approach allows for early detection of metabolic abnormalities and timely therapeutic intervention.

In the presented familial case, marked clinical heterogeneity of 17q12 microdeletion syndrome is evident, with predominant neuropsychiatric manifestations observed in the daughter.

To compare the clinical features observed in this family with those characteristic of classical MODY5, we performed a comparative analysis with a previously published case carrying the pathogenic variant c.826C>T in the *HNF1B* gene, resulting in a premature stop codon (p.Arg276Ter) and loss of transcription factor function (11, 12).

## Clinical and laboratory characteristics of a patient carrying a pathogenic *HNF1B* variant

**Figure 2:**
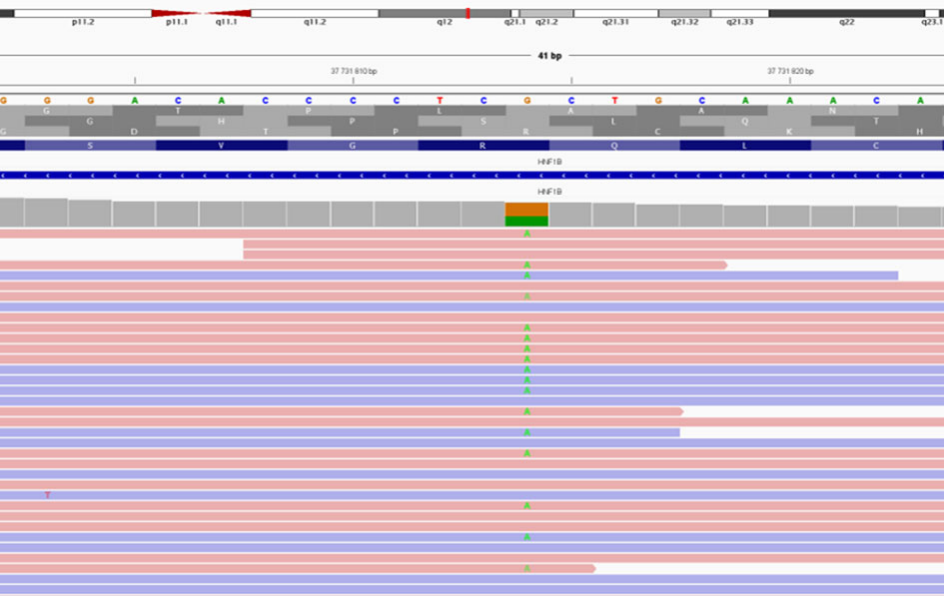
Targeted next-generation sequencing (NGS), results identifying a pathogenic variant c.826C>T in the HNF1B gene (hg19), resulting in a premature stop codon (p.Arg276Ter) and loss of transcription factor function.

At the age of 15 years, during a routine clinical evaluation, renal ultrasound revealed bilaterally reduced kidney size with the presence of multiple cysts in both kidneys. Laboratory testing demonstrated impaired renal function, with a serum creatinine level of 114.7 µmol/L and an eGFR of 77 mL/min/1.73 m², calculated using the Schwartz formula. Blood biochemistry revealed hyperphosphatemia (up to 2.28 mmol/L) and elevated uric acid levels (up to 476.7 µmol/L). Molecular genetic testing using a targeted gene panel for hereditary forms of diabetes was subsequently performed (**Figure 2**).

During inpatient evaluation, fasting plasma glucose levels ranged from 5.2 to 6.6 mmol/L, with postprandial values reaching up to 6.8 mmol/L at 2 h after meals. HbA1c level was 6.1%. A standard 75-g oral glucose tolerance test demonstrated impaired glucose tolerance and insulin resistance (HOMA-IR = 3.75), while endogenous insulin secretion remained preserved (basal C-peptide 3.41 ng/mL). Dietary therapy with regular monitoring of carbohydrate metabolism parameters was recommended. Further examination confirmed progression of renal dysfunction, with a decrease in eGFR to 53 mL/min/1.73 m² (Schwartz formula). Persistent hyperuricemia (up to 519.4 µmol/L), elevated inorganic phosphorus levels (1.91 mmol/L), and low-normal serum magnesium concentration (0.73 mmol/L) were also observed. The complete results of laboratory and instrumental investigations are presented in **Supplementary 5**.

Overall, the clinical and laboratory findings in this patient indicate a more pronounced decline in renal filtration function, accompanied by higher serum phosphorus and uric acid levels, consistent with the renal and metabolic manifestations associated with pathogenic HNF1B variants.

## Discussion

Currently, a limited number of publications describe the clinical and laboratory characteristics of patients with 17q12 recurrent deletion syndrome. Existing studies emphasize both intrafamilial phenotypic variability and marked interfamilial heterogeneity among individuals carrying a 17q12 microdeletion (5).

In the presence of a deletion of the same size, an extremely diverse clinical picture can be observed in different patients, ranging from asymptomatic carriage to severe multisystem disease in children or even fatal renal agenesis in fetuses. A recent paper by Italian authors describes three familial cases with significant variability in clinical and laboratory parameters, ranging from minor neuropsychiatric developmental disorders to macrocephaly with delayed cognitive and motor development and febrile seizures (13).

Chinese colleagues presented an interesting case of confirmed recurrent 17q12 deletion syndrome in a 25-year-old patient with low-grade serous ovarian carcinoma (stage IIIC). The patient’s mother had structural and functional changes suggestive of a 17q12 deletion, including diabetes mellitus, a single kidney, a partially septate uterus, and hemochromatosis. These features could represent manifestations of the deletion syndrome or concomitant pathology; however, CMA could not be performed due to the mother’s early death (14).

There is also evidence associating 17q12 deletion with Mayer−Rokitansky−Küster−Hauser syndrome. Two sporadic cases of unrelated Italian patients carrying a deletion of approximately 1.5 Mb, with variable phenotypes, have been described. The first patient presented with dysmorphic features, including sparse lateral eyebrows, obliquely positioned palpebral fissures, hypertrichosis of the upper lip, congenital absence of the uterus, polycystic left ovary, a probable remnant of the left Müllerian duct, and no renal pathology. The second patient had polycystic kidney disease, agenesis of the upper and middle thirds of the vagina, and a right-sided unicornuate uterus. No disturbances of carbohydrate, purine, or calcium−phosphorus metabolism were detected (15).

A familial case of recurrent 17q12 deletion from New Zealand with possible incomplete penetrance and variable expressivity has been reported. In that family, the mother — a presumed carrier of the deletion — had no carbohydrate metabolism disorders or renal morphological changes but had learning difficulties and required special needs classes at school. The index patient was diagnosed with attention−deficit/hyperactivity disorder, destructive behavior, and learning difficulties, while her general cognitive abilities were within the “lower middle range” of intellectual function. Ultrasound examination revealed no urinary tract abnormalities. Her 2.5−year−old brother had mild developmental delay and bilateral cystic kidney disease; he exhibited isolated mild delay in gross motor skills and began sitting at 9 months of age (16).

In a familial case reported by colleagues from Turkey, there was a marked difference in the clinical presentation of diabetes mellitus and the required antidiabetic treatment. The index patient, with a CMA−confirmed 17q12 deletion, presented with diabetic ketoacidosis at a young age, had a stable C−peptide level, and subsequently required insulin therapy. In contrast, the patient’s mother developed diabetes at the age of 30 and was successfully treated with oral glucose−lowering agents. Interfamilial variability may reflect tissue-specific sensitivity to reduced gene dosage, whereas genetic modifiers, epigenetic factors, and environmental exposures contributing to phenotypic diversity likely influence intrafamilial variability (17).

Wu et al. demonstrated that retinopathy, pancreatic dysplasia with diabetes, and neurodevelopmental disorders are associated with haploinsufficiency of several genes located within the 17q12 region, including *HNF1B, LHX1, ACACA, PIGW*, and *MIR2909*. In addition, clinical heterogeneity may also be influenced by haploinsufficiency of *TBC1D3* and its paralogs. Expression of *TBC1D3* has been shown to delay ubiquitination and degradation of insulin receptor substrate-1 (IRS-1), potentially disrupting insulin signaling pathways and contributing to insulin resistance or diabetes mellitus through dysregulated IRS-1 turnover in insulin-responsive tissues (18). In the present family, the 1.4-Mb deleted segment encompasses 15 genes (*AATF, ACACA, C17orf78, DDX52, DHRS11, DUSP1, GGNBP2, HNF1B, LHX1, MRM1, MYO19, PIGW, SYNRG*, and *TADA2A*), each of which may contribute to the observed clinical variability.

Analysis of chromosomal alterations affecting the 17q12 region supports the notion that *HNF1B* plays a central role in the development of the majority of renal and metabolic features associated with 17q12 microdeletion and microduplication syndromes (19). Nevertheless, the broader phenotypic spectrum observed in deletion carriers likely reflects the combined effects of multiple dosage-sensitive genes within this locus.

To date, more than 400 pathogenic variants in the *HNF1B* gene have been identified in patients with maturity-onset diabetes of the young type 5 (MODY5), with *de novo* mutations accounting for approximately 30-50% of cases. These variants include missense, nonsense, frameshift, splice-site mutations, small insertions and deletions, as well as large genomic deletions (20). Intragenic variants are most frequently located within the DNA-binding domain (exons 2 and 4), while whole-gene deletions account for up to 50% of pathogenic *HNF1B* alterations (8). Carriers of *HNF1B* variants exhibit substantial phenotypic heterogeneity both between and within families. No consistent genotype–phenotype correlation has been established, and haploinsufficiency is considered the principal pathogenic mechanism.

In most individuals with pathogenic *HNF1B* variants, renal cystic disease and impaired renal function are common early manifestations and often precede disturbances of carbohydrate metabolism. In addition to diabetes and renal abnormalities, affected patients may present with a wide range of extrarenal features, including ophthalmological disorders (hypermetropia, cataracts, retinopathy), congenital heart defects, anomalies of the reproductive system (vaginal aplasia, uterine malformations, epididymal cysts, vas deferens atresia), hepatic cysts, and pancreatic abnormalities such as atrophy, agenesis, or calcifications (21, 22). Metabolic disturbances, including elevated liver enzymes (ALT, AST, GGT), hypomagnesemia, hypokalemia, hyperuricemia, and hyperparathyroidism, have also been reported (22).

Given the overlapping but non-identical phenotypic spectra, an important clinical question is whether patients with a 17q12 deletion differ systematically from those carrying intragenic *HNF1B* variants. According to Buffin-Meyer et al., progression to chronic kidney disease and the occurrence of end-stage renal disease were less frequent among patients with a 17q12 deletion than among carriers of pathogenic *HNF1B* variants. Within the *HNF1B* variant group, slower progression to CKD stage 3 was observed in patients with variants affecting the POU homeodomain compared with those involving the POU-specific domain. With regard to extrarenal manifestations, 17q12 microdeletion carriers demonstrated a higher prevalence of hypomagnesemia and neurodevelopmental disorders but a lower frequency of hyperuricemia. No statistically significant difference in hyperglycemia prevalence was detected, although a trend toward lower frequency was noted in the deletion group (23).

Cannon et al., in a study assessing the pathogenic effects of 17q12 copy number variations, reported that impaired carbohydrate metabolism was specifically associated with microdeletions rather than microduplications. Renal involvement was observed in both groups; however, individuals with microdeletions exhibited a lower estimated glomerular filtration rate. Furthermore, microdeletions were associated with intellectual disability, whereas microduplications were more frequently linked to psychiatric disorders (24). In a comparative analysis by Clissold et al. involving 18 patients with intragenic *HNF1B* mutations and 20 patients with a 17q12 deletion, autistic traits and higher levels of psychopathology were more prevalent in the deletion group, while no differences in cognitive abilities were observed (25). Consistent with these findings, a recent large-scale study of CNV loci in neuropsychiatric disorders demonstrated that the 17q12 microdeletion was associated with the highest risk for the development of autism spectrum disorder among all CNVs analyzed (26).

Differences in neurodevelopmental and neuropsychiatric manifestations observed in patients with 17q12 microdeletion syndrome may be related to the loss of gene products encoded within the deleted region. For example, the *LHX1* gene plays an essential role in neuronal differentiation, axonal migration, and cerebellar development; therefore, reduced gene dosage may contribute to the development of ASD and other disorders of the nervous system. The gametogenetin-binding protein 2 (*GGNBP2*) gene has also been included among candidate genes associated with ASD and is listed in autism-related genetic databases (19).

Another potential mechanism underlying increased susceptibility to neuropsychiatric disorders involves epigenetic alterations. Previous studies have reported changes in DNA methylation patterns associated with 17q12 deletions, including hypomethylation of *SLC1A3* on chromosome 5, a gene whose variants have been implicated in ASD and schizophrenia. Such epigenetic dysregulation may further modulate phenotypic variability in affected individuals (27).

Given the broad phenotypic spectrum associated with pathogenic alterations in the 17q12 region, further research is required to optimize personalized approaches to treatment, monitoring, and prevention of complications. Cui et al., who generated induced pluripotent stem cells, undertook an important step toward individualized disease modeling (PLAFMCi005-A) by reprogramming peripheral blood mononuclear cells from a patient carrying a pathogenic *HNF1B* splice-site variant (NM_000458:exon2:c.544 + 3_544 + 6delAAGT). This model provides a valuable platform for studying RCAD syndrome associated with *HNF1B* mutations and may facilitate deeper insight into disease mechanisms and translational applications (28, 29).

Furthermore, the use of proteomic approaches could significantly deepen our understanding of the functional impact of deletions in the 17q12 region, allowing for more accurate modeling of biological processes, investigation of disease mechanisms, and development of novel therapeutic strategies. Proteomic and bioinformatic analysis of tissues with variable sizes of chromosome 17q12 deletions and different spectra of lost genes will enable the identification of a broader range of targets and downstream signaling pathways. This will provide a more detailed picture of the pathogenic molecular mechanisms underlying the disease and expand the possibilities for monitoring disease stages, investigating disease progression, and selecting appropriate treatment modalities (30).

The familial case of 17q12 recurrent deletion syndrome described in the present study confirms pronounced intrafamilial clinical variability and expands the existing clinical characterization of this condition through detailed laboratory and instrumental assessments.

## Study limitations

The unavailability of the index patient’s father precluded definitive assessment of inheritance patterns, limiting the ability to distinguish between inherited and *de novo* occurrence of the 17q12 deletion. Differences in clinical manifestations, including the presence of ASD in the patient’s daughter, may reflect the contribution of additional genetic variants or modifying factors located in noncoding or regulatory genomic regions. Addressing these possibilities would require whole-genome sequencing and systematic analysis of additional genetic modifiers. Furthermore, investigation of epigenetic alterations may provide additional insight into mechanisms underlying phenotypic variability.

## Conclusion

In this study, we describe, to our knowledge, the first reported familial case of recurrent chromosome 17q12 deletion syndrome in a Russian family. The available data suggest that the deletion in the index patient most likely arose *de novo*, consistent with the known mutational mechanisms underlying this condition. The identified deletion expands the spectrum of molecular alterations associated with 17q12 deletion syndrome and contributes to existing genotype data.

Detailed clinical and laboratory characterization of the affected family members enhances current understanding of phenotypic variability associated with pathogenic alterations in the 17q12 region. The presented cases are consistent with previously published data and illustrate marked differences in clinical and laboratory parameters, including more pronounced renal dysfunction and hyperuricemia in a patient with an intragenic pathogenic *HNF1B* variant, as well as hypomagnesemia and neurodevelopmental manifestations in patients with 17q12 microdeletion syndrome.

## Data Availability

The original contributions presented in the study are included in the article/**Supplementary Material**. Further inquiries can be directed to the corresponding author/s.
