# Supplementary material for: A familial case report of 17q12 recurrent deletion syndrome: clinical and molecular characterization
Source: Front Endocrinol (Lausanne). 2026 May 12;17:1819047. doi: 10.3389/fendo.2026.1819047 (PMC13201108; doi:10.3389/fendo.2026.1819047)
Supplement: Supplementary file 1 [file Table1.docx]

Supplementary 1. Chronologically structured profile of glycated hemoglobin (HbA1c), C-peptide, and insulin levels during the 14-year follow-up of the patient.

| Date | HbA1c, % | C-peptide, ng/mL | Insulin, µU/mL | Treatment |
| --- | --- | --- | --- | --- |
| 05.11.2011 | 7.1 | – | – | Diet therapy |
| 28.12.2011 | – | 2.4 | – | Diet therapy |
| 15.01.2012 | – | – | <2 | Diet therapy |
| 30.01.2012 | 6.7 | 0.765 | – | Diet therapy |
| 14.05.2012 | 6.4 | – | – | Diet therapy |
| 06.03.2012 | – | 1.01 | 1.8 | Diet therapy |
| 16.04.2012 | – | 1.22 | 4.5 | Diet therapy |
| 14.05.2012 | – | 1.119 | 7.4 | Diet therapy |
| 02.08.2012 | 6.5 | 1.04 | 4.9 | Diet therapy |
| 30.10.2012 | 6 | – | – | Diet therapy |
| 20.12.2012 | 5.8 | – | – | Diet therapy |
| 21.03.2013 | 6 | – | – | Diet therapy |
| 25.06.2013 | 6 | – | – | Diet therapy |
| 19.08.2013 | 5.8 | – | – | Diet therapy |
| 22.11.2013 | 5.7 | – | – | Diet therapy |
| 27.01.2014 (Pregnancy) | 5.4 | – | – | Diet therapy |
| 26.02.2014 (Pregnancy) | 5.5 | – | – | Diet therapy |
| 25.04.2014 (Pregnancy) | 5.4 | – | – | Diet therapy |
| 12.05.2014 (Pregnancy) | 5.3 | – | – | Diet therapy |
| 27.08.2014 | 5.2 | – | – | Diet therapy |
| 04.12.2014 | 5.9 | – | – | Diet therapy |
| 04.12.2014 | 6 | – | 4.1 | Diet therapy |
| 16.03.2015 | 6.3 | – | – | Diet therapy |
| 03.06.2015 | 5.2 | – | – | Diet therapy |
| 10.07.2015 | 5.7 | 0.7 | – | Diet therapy |
| 28.08.2015 | 5.3 | – | – | Diet therapy |
| 23.05.2016 | 5.5 | 0.7 | – | Diet therapy |
| 17.11.2016 | 6 | 0.89 | – | Diet therapy |
| 28.03.2017 | 5.9 | 1.18 | – | Diet therapy |
| 21.08.2017 | 5.8 | 1.04 | – | Diet therapy |
| 24.11.2017 (Pregnancy) | 5.2 | – | – | Diet therapy |
| 01.02.2018 (Pregnancy) | 5.2 | – | – | Diet therapy |
| 12.03.2018 (Pregnancy) | 5.3 | – | – | Long-acting insulin |
| 03.04.2018 (Pregnancy) | 5.0 | – | – | Long-acting insulin |
| 21.08.2018 | 6.2 | – | – | Diet therapy |
| 04.02.2019 | 6.4 | – | – | Diet therapy |
| 17.08.2019 | 7.2 | 1.33 | – | Diet therapy |
| 23.09.2020 | 6.6 | – | – | Diet therapy |
| 02.06.2021 (Pregnancy) | 6.9 | – | – | Long-acting insulin |
| 26.07.2021 (Pregnancy) | 6 | – | – | Long-acting insulin |
| 28.09.2021 (Pregnancy) | 6.5 | – | – | Long-acting + short-acting insulin |
| 29.10.2021 (Pregnancy) | 6.4 | – | – | Long-acting + short-acting insulin |
| 26.12.2021 (Pregnancy) | 6.2 | – | – | Long-acting + short-acting insulin |
| 09.06.2022 | 7.86 | – | – | Diet therapy |
| 10.02.2023 | 8.1 | – | – | Diet therapy |
| 09.06.2023 | 8.5 | 2.17 (postprandial) | – | Diet therapy |
| 22.03.2024 | 6.1 | – | – | Diet therapy |
| 12.12.2024 | 8.1 | 1.8 | – | Diet therapy |

Supplementary 2**.** Laboratory examination results of the index patient. Values above or below the reference range are highlighted in bold.

| \| **Glycated hemoglobin** \| \| --- \| \| Glycated hemoglobin**(**NGSP) **8.7**  % (4 - 6) \| \| Glycated hemoglobin  (IFCC) **72**  mmol/mol (20 - 43) \| \| Estimated Average Glucose (eAG) 11.2 mmol/l \| \|  \| \| **Complete Blood Count** \| \|  \| \| Leukocytes 7.09 10^9^ cells/l  (3.4 - 10.8) \| \| - Neutrophils **37.6**  % (39 - 75.0) \| \| - Lymphocytes **51.1**  % (19 - 39.0) \| \| - Monocytes 7.5 % (3 - 11.0) \| \| - Eosinophils  3.5 % (0.2 - 5.4) \| \| - Basophils 0.3 % (0 - 1) \| \| - Neutrophils (abs) 2.67 10^9^ cells/l  (1.5 - 6.8) \| \| - Lymphocytes (abs) 3.62 10^9^ cells/l  (0.86 - 4.07) \| \| - Monocytes (abs) 0.53 10^9^ cells/l  (0.3 - 1) \| \| - Eosinophils (аbs) 0.25 10^9^ cells/l  (0 - 0.4) \| \| - Basophils (аbs) 0.02 10^9^ cells/l  (0 - 0.1) \| \| Erythrocytes 4.82 10^12^ cells/l  (3.8 - 5.2) \| \| Hemoglobin 136 g/l  (112 - 153) \| \| Hematocrit (%) 41.1 % (35 - 46) \| \| Mean Corpuscular Volume 85  fl  (82 - 98) \| \| Mean Corpuscular Hemoglobin 28.2 pg (27 - 34) \| \| Mean Corpuscular Hemoglobin Concentration 331 g/l  (314 - 356) \| \| Red Cell Distribution Width 13.2 % (11.6 - 16.5) \| \| Platelets 334 10^9^ cells/l  (152 - 372) \| \| Mean Platelet Volume  10.1  fl  (9 - 12.2) \| \| Plateletcrit 0.34 % (0.17 - 0.38) \| \| Platelet Distribution Width 11.0 % (9.7 - 16.7) \| \| ESR 3 mm/hour  (2 - 20) \| \|  \| \| **Blood Chemistry Analysis** \| \| Glucose **7.7**  mmol/l (3.1 - 6.1) \| \| Sodium 139.3 mmol/l (135 - 145) \| \| Potassium 3.77 mmol/l (3.5 - 5.1) \| \| Chloride 100.1 mmol/l (98 - 107) \| \| Magnesium **0.49**  mmol/l (0.7 - 1.05) \| \| Total Calcium 2.22 mmol/l (2.15 - 2.55) \| \| Phosphorus 1.19 mmol/l (0.74 - 1.52) \| \| Total Protein 76.8 g/l  (64 - 83) \| \| Albumin 42.4 g/l  (35 - 50) \| \| Urea **7.3**  mmol/l (2.5 - 6.7) \| \| Creatinine 79.5 mmol/l (50 - 98) \| \| eGFR (CKD-EPI) = 80 mL/min/1,73 m^2^ \| \| Uric Acid 257 µmol/l (142 - 339) \| \| Total Bilirubin 15.8 µmol/l (3.4 - 20.5) \| \| Aspartate Aminotransferase (AST) **48.4**   U/l (5 - 34) \| \| Alanine Aminotransferase (ALT) 41.2  U/l (0 - 55) \| \| Cholesterol  **5.3**  mmol/l (3.3 - 5.2) \| \| LDL Cholesterol **3.34**  mmol/l (1.1 - 3) \| \| HDL Cholesterol 1.56 mmol/l (1.15 - 2.6) \| \| Triglycerides 0.9 mmol/l (0.1 - 1.7) \| \|  \| \| **Hormonal Blood Analysis** \| \| C-peptide 1.31 ng/ml (1.1 - 4.4) \| \| Thyroid-Stimulating Hormone (TSH) 1.876 mU/l (0.25 - 3.5) \| \| Parathyroid Hormone (PTH) 41.5 pg/ml (15 - 65) \| \|  \| \| **General Urine Analysis** \| \|  \| \| Physico-chemical properties \| \| Glucose 0 mmol/l (0 - 2.8) \| \| Protein  0 g/l  (0 - 0.2) \| \| Bilirubin 0 µmol/l (0 - 10) \| \| Urobilinogen  0 µmol/l (0 - 34) \| \| Acidity (pH) 6 - (5 - 6) \| \| Erythrocytes (hemoglobin) 0 in µl (0 - 10) \| \| Ketones 0 mmol/l (0 - 0.5) \| \| Nitrites Not detected \| \| Leukocytes  0 in 1 µl  (0 - 25) \| \| Transparency Clear Clear \| \| Relative Density  **1.008**  g/l  (1.02 - 1.03) \| \| Color Light yellow \| \|  \| \| **Urine Biochemistry Analysis** \| \| Albumin/creatinine ratio in a random urine sample \| \|  \| \| Creatinine 5045 µmol/l  (2470- 19200) \| \| Albumin 2.0 mg/l (0 - 20) \| \| Albumin/Creatinine 0.40 mg/mmol  (0 - 3.5) \| |
| --- | --- | --- | --- | --- | --- | --- | --- | --- | --- | --- | --- | --- | --- | --- | --- | --- | --- | --- | --- | --- | --- | --- | --- | --- | --- | --- | --- | --- | --- | --- | --- | --- | --- | --- | --- | --- | --- | --- | --- | --- | --- | --- | --- | --- | --- | --- | --- | --- | --- | --- | --- | --- | --- | --- | --- | --- | --- | --- | --- | --- | --- | --- | --- | --- | --- | --- | --- | --- | --- | --- | --- | --- | --- | --- | --- | --- | --- | --- | --- | --- |

| **Renal Ultrasound** | |
| --- | --- |
| Right kidney | Contours are smooth. Located typically. Mobility: no pathological mobility detected. Dimensions: length: 10 cm, width: 4.9 cm, thickness: 5.8 cm. Parenchyma is heterogeneous. Echogenicity is within normal limits. Parenchymal thickness: 1.8 cm. The pelvicalyceal system is not compacted or deformed. No calculi detected. Space-occupying lesions: anechoic formations are identified in the parenchyma, measuring 0.9 cm in the upper third and 1.4 cm in the lower third. |
| Left kidney | Contours are smooth. Located typically. Mobility: no pathological mobility detected. Dimensions: length: 10.7 cm, width: 4.9 cm, thickness: 4.6 cm. Parenchyma is heterogeneous. Echogenicity is within normal limits. Parenchymal thickness: 1.7 cm. The pelvicalyceal system is not compacted or deformed. No calculi detected. Space-occupying lesions: multiple anechoic formations are present in the parenchyma, measuring 0.6 cm in the upper third and up to 1.5 cm in the lower third. |
| **Abdominal ultrasound** | |
| Liver | Location is typical. Contours are smooth and clear. Dimensions: right lobe thickness: 10 cm, left lobe thickness: 5.5 cm. Portal vein diameter: 1.1 cm. Intrahepatic bile ducts are not dilated. Common bile duct is not dilated. Parenchymal structure is homogeneous. Echogenicity is unchanged. No space-occupying lesions are identified |
| Gallbladder | Contours are smooth and clear. Dimensions: length: 7.2 cm, thickness: 3.7 cm. Content is homogeneous. Walls are not compacted or thickened; wall thickness: 0.2 cm. No calculi detected. No lesions identified. |
| Pancreas | Contours: the tail is obscured by bowel gas. Dimensions (thickness): head: 1.6 cm, body: 1.2 cm. Structure is heterogeneous. Echogenicity is equal to that of the liver. Wirsung's duct (pancreatic duct) is not dilated. No lesions identified. |
| Spleen | Contours are smooth and clear. Location is typical. Dimensions: length: 9.7 cm, thickness: 4.2 cm. Parenchymal structure is homogeneous. Echogenicity is unchanged. Splenic vein diameter: 0.6 cm. No lesions identified. |

Supplementary 3. Laboratory Monitoring Parameters Prior to Hospitalization. Values outside the reference range are highlighted in bold.

| Parameter | Date | | |
| --- | --- | --- | --- |
|  | 11.2022 | 06.2023 | 03.2024 |
| 25-(ОН) vitamin D, ng/ml (30-75) | **7,4** | **29,7** | **28,3** |
| Sodium, mmol/l  (136-146) | 137,3 | 135,6 | 140,3 |
| Uric Acid, µmol/l  (154,7-357) | 222 | 202,6 | 244,8 |
| Potassium, mmol/l  (3,5-5,1) | **5,29** | 4,41 | 4,7 |
| Phosphorus, mmol/l  (1,29-2,26) | 1,49 | 1,33 | 1,58 |
| Ionized calcium, mmol/l  (1,05-1,3) | 1,15 | 1,15 | 1,15 |
| Creatinine µmol/l  (23-68) | 28,7 | 33,3 | 33,8 |
| Total Calcium, mmol/l  (2,08-2,65) | 2,55 | 2,49 | 2,53 |
| Urea, mmol/l  (1,8-6,4) | **7,5** | 5,5 | **9,2** |
| Alkaline phosphatase, U/l  (51-332) | **348,7** | **360,4** | **411,9** |
| Parathyroid Hormone,  pmol/l  (1,7-7,63) | 4,0 | 5,1 | 7,49 |

Supplementary 4. Laboratory Parameters of the Index Patient's Elder Daughter. Values outside the reference range are highlighted in bold.

| **Glycated hemoglobin** |
| --- |
| Glycated hemoglobin (NGSP) 5.5 % (4 - 6) |
|  |
| **Blood Chemistry Analysis** |
| Urea 5.59 mmol/l (1.8 - 6.4) |
| Total Bilirubin 10.86 µmol/l (2 - 15.6) |
| Aspartate Aminotransferase (AST) **49.7**  U/l (13 - 35) |
| Alanine Aminotransferase (ALT) 33.1 U/l (7 - 35) |
| Gamma-glutamyl transpeptidase (GGT) 12.6 U/l (4-12) |
| Creatinine 38.7 µmol/l (27 - 62) |
| Glucose 4.46 mmol/l (3.1 – 6.3) |
| Magnesium 0.73 mmol/l (0.7 – 1.05) |
| Total Calcium 2.48 mmol/l (2.15 - 2.55) |
| Albumin 44 g/l  (35 – 50) |
| Sodium 133.5 mmol/l (135 - 145) |
| Potassium 4.75 mmol/l (3.5 – 5.1) |
| Chloride 100.5 mmol/l (98 - 107) |
| Alkaline phosphatase 433.4 mmol/l (51 – 332) |
| 25-(ОН) vitamin D 32.7 ng/ml (30 – 75) |
|  |
| **Hormonal Blood Analysis** |
| C-peptide 1.08 ng/ml (1 - 4.4) |
| Parathyroid Hormone (PTH) 40.4 pg/ml (15 - 65) |
|  |
| **General Urine Analysis** |
|  |
| Glucose 0 mmol/l (0 - 2.8) |
| Protein  0 g/l  (0 - 0.2) |
| Bilirubin 0 µmol/l (0 - 10) |
| Urobilinogen  0 µmol/l (0 - 34) |
| Acidity (pH) 5.5 - (5 - 6) |
| Erythrocytes (hemoglobin) 0 in µl (0 - 10) |
| Ketones 0 mmol/l (0 - 0.5) |
| Nitrites Not detected |
| Leukocytes  0 in 1 µl  (0 - 25) |
| Transparency Clear Clear |
| Relative Density  1.009 g/l  (1.008-1.025) |
| Color Light yellow |
| Glucose 0 mmol/l (0 - 2.8) |

| **Renal Ultrasound** | |
| --- | --- |
| Right Kidney | Position: Usual. Dimensions: 85 x 36 x 41 mm. Architecture: Differentiation is preserved. Echogenicity: Slightly increased. Parenchymal Findings: Anechoic inclusions measuring 3.5 mm and 3.0 mm. Pelvicalyceal System: Not dilated. |
| Left Kidney | Position: Usual. Dimensions: 88 x 35 x 41 mm. Architecture: Differentiation is preserved. Echogenicity: Slightly increased. Parenchymal Findings: Two small anechoic formations measuring 3.2 mm and 3.3 mm. Additionally, a poorly visualized anechoic, avascular (on color Doppler imaging) formation measuring 10 x 9 mm is seen in the lower third. Pelvicalyceal System: The renal pelvis is dilated, measuring up to 14.2 mm (pyelectasis). |
| Conclusion | Ultrasound findings are consistent with polycystic kidney disease. Pyelectasis of the left kidney. |
| **Ultrasound of the Urinary Bladder with Post-Void Residual Measurement** | |
| Urinary Bladder | Filling Volume: Approximately 160 ml. Contours: Clear and smooth. Wall Thickness: 2.5 mm. Content: Anechoic, no sediment. Post-Void Residual Urine: 8 ml. |
| Conclusion | Ultrasound findings show no structural changes. |

Supplementary 5. Laboratory Parameters of the Patient with an HNF1B Gene Variant. Values outside the reference range are highlighted in bold.

| \|  \|  \|  \|  \|  \|  \|  \|  \|  \|  \|  \|  \|  \|  \|  \|  \|  \|  \|  \|  \|  \|  \|  \|  \|  \|  \|  \|  \|  \|  \|  \|  \|  \|  \|  \|  \|  \|  \|  \|  \|  \|  \|  \|  \|  \|  \|  \|  \|  \|  \| \| --- \| --- \| --- \| --- \| --- \| --- \| --- \| --- \| --- \| --- \| --- \| --- \| --- \| --- \| --- \| --- \| --- \| --- \| --- \| --- \| --- \| --- \| --- \| --- \| --- \| --- \| --- \| --- \| --- \| --- \| --- \| --- \| --- \| --- \| --- \| --- \| --- \| --- \| --- \| --- \| --- \| --- \| --- \| --- \| --- \| --- \| --- \| --- \| --- \| --- \| \| Glycated hemoglobin (NGSP) \| \| \| \| \| \| \| \| \| \| \| \| \| \| \| \| \| \| \| **6.1** \| \| \| \| \| \| \| \| \| \| % \| \| \| \| \| \| \| \| \| \| (4.0 - 6.0) \| \| \| \| \| \| \| \| \| \| \| \| Glycated hemoglobin (IFCC) \| \| \| \| \| \| \| \| \| \| \| \| \| \| \| \| \| \| \| 43 \| \| \| \| \| \| \| \| \| \| mmol/mol \| \| \| \| \| \| \| \| \| \| (20 - 43) \| \| \| \| \| \| \| \| \| \| \| \| Estimated Average Glucose (eAG) \| \| \| \| \| \| \| \| \| \| \| \| \| \| \| \| \| \| \| 7.1 \| \| \| \| \| \| \| \| \| \| mmol/l \| \| \| \| \| \| \| \| \| \| \| \| \| \| \| \| \| \| \| \| \| | | | |
| --- | --- | --- | --- | --- | --- | --- | --- | --- | --- | --- | --- | --- | --- | --- | --- | --- | --- | --- | --- | --- | --- | --- | --- | --- | --- | --- | --- | --- | --- | --- | --- | --- | --- | --- | --- | --- | --- | --- | --- | --- | --- | --- | --- | --- | --- | --- | --- | --- | --- | --- | --- | --- | --- | --- | --- | --- | --- | --- | --- | --- | --- | --- | --- | --- | --- | --- | --- | --- | --- | --- | --- | --- | --- | --- | --- | --- | --- | --- | --- | --- | --- | --- | --- | --- | --- | --- | --- | --- | --- | --- | --- | --- | --- | --- | --- | --- | --- | --- | --- | --- | --- | --- | --- | --- | --- | --- | --- | --- | --- | --- | --- | --- | --- | --- | --- | --- | --- | --- | --- | --- | --- | --- | --- | --- | --- | --- | --- | --- | --- | --- | --- | --- | --- | --- | --- | --- | --- | --- | --- | --- | --- | --- | --- | --- | --- | --- | --- | --- | --- | --- | --- | --- | --- | --- | --- | --- | --- | --- | --- | --- | --- | --- | --- | --- | --- | --- | --- | --- | --- | --- | --- | --- | --- | --- | --- | --- | --- | --- | --- | --- | --- | --- | --- | --- | --- | --- | --- | --- | --- | --- | --- | --- | --- | --- | --- | --- | --- | --- | --- | --- | --- | --- | --- |
| \| **Complete Blood Count** \| \| --- \|  \|  \|  \|  \|  \|  \|  \|  \|  \|  \|  \|  \|  \|  \|  \|  \|  \|  \|  \|  \|  \|  \|  \|  \|  \|  \|  \|  \|  \|  \|  \|  \|  \|  \|  \|  \|  \|  \|  \|  \|  \|  \|  \|  \|  \|  \|  \|  \|  \|  \|  \| \| --- \| --- \| --- \| --- \| --- \| --- \| --- \| --- \| --- \| --- \| --- \| --- \| --- \| --- \| --- \| --- \| --- \| --- \| --- \| --- \| --- \| --- \| --- \| --- \| --- \| --- \| --- \| --- \| --- \| --- \| --- \| --- \| --- \| --- \| --- \| --- \| --- \| --- \| --- \| --- \| --- \| --- \| --- \| --- \| --- \| --- \| --- \| --- \| --- \| --- \| \| Leukocytes \| \| \| \| \| \| \| \| \| \| \| \| \| \| \| \| \| \| \| **4.29** \| \| \| \| \| \| \| \| \| \| 10^9^ cells/l \| \| \| \| \| \| \| \| \| \| (4.50 - 9.50) \| \| \| \| \| \| \| \| \| \| \| \| - Neutrophils \| \| \| \| \| \| \| \| \| \| \| \| \| \| \| \| \| \| \| **60.4** \| \| \| \| \| \| \| \| \| \| % \| \| \| \| \| \| \| \| \| \| (40.0 - 60.0) \| \| \| \| \| \| \| \| \| \| \| \| - Lymphocytes \| \| \| \| \| \| \| \| \| \| \| \| \| \| \| \| \| \| \| 31.8 \| \| \| \| \| \| \| \| \| \| % \| \| \| \| \| \| \| \| \| \| (19.0 - 39.0) \| \| \| \| \| \| \| \| \| \| \| \| - Monocytes \| \| \| \| \| \| \| \| \| \| \| \| \| \| \| \| \| \| \| 6.6 \| \| \| \| \| \| \| \| \| \| % \| \| \| \| \| \| \| \| \| \| (1.0 - 11.0) \| \| \| \| \| \| \| \| \| \| \| \| - Eosinophils \| \| \| \| \| \| \| \| \| \| \| \| \| \| \| \| \| \| \| 1.2 \| \| \| \| \| \| \| \| \| \| % \| \| \| \| \| \| \| \| \| \| (0.2 - 5.4) \| \| \| \| \| \| \| \| \| \| \| \| - Basophils \| \| \| \| \| \| \| \| \| \| \| \| \| \| \| \| \| \| \| 0.0 \| \| \| \| \| \| \| \| \| \| % \| \| \| \| \| \| \| \| \| \| (0.0 - 1.0) \| \| \| \| \| \| \| \| \| \| \| \| - Neutrophils (abs) \| \| \| \| \| \| \| \| \| \| \| \| \| \| \| \| \| \| \| 2.60 \| \| \| \| \| \| \| \| \| \| 10^9^ cells/l \| \| \| \| \| \| \| \| \| \| (1.50 - 6.80) \| \| \| \| \| \| \| \| \| \| \| \| - Lymphocytes (abs) \| \| \| \| \| \| \| \| \| \| \| \| \| \| \| \| \| \| \| 1.36 \| \| \| \| \| \| \| \| \| \| 10^9^ cells/l \| \| \| \| \| \| \| \| \| \| (0.86 - 4.07) \| \| \| \| \| \| \| \| \| \| \| \| - Monocytes (abs) \| \| \| \| \| \| \| \| \| \| \| \| \| \| \| \| \| \| \| 0.28 \| \| \| \| \| \| \| \| \| \| 10^9^ cells/l \| \| \| \| \| \| \| \| \| \| (0.00 - 0.80) \| \| \| \| \| \| \| \| \| \| \| \| - Eosinophils (abs) \| \| \| \| \| \| \| \| \| \| \| \| \| \| \| \| \| \| \| 0.05 \| \| \| \| \| \| \| \| \| \| 10^9^ cells/l \| \| \| \| \| \| \| \| \| \| (0.00 - 0.40) \| \| \| \| \| \| \| \| \| \| \| \| - Basophils (abs) \| \| \| \| \| \| \| \| \| \| \| \| \| \| \| \| \| \| \| 0.00 \| \| \| \| \| \| \| \| \| \| 10^9^ cells/l \| \| \| \| \| \| \| \| \| \| (0.00 - 0.10) \| \| \| \| \| \| \| \| \| \| \| \| Erythrocytes \| \| \| \| \| \| \| \| \| \| \| \| \| \| \| \| \| \| \| 5.66 \| \| \| \| \| \| \| \| \| \| 10^12^ cells/l \| \| \| \| \| \| \| \| \| \| (4.30 - 5.80) \| \| \| \| \| \| \| \| \| \| \| \| Hemoglobin \| \| \| \| \| \| \| \| \| \| \| \| \| \| \| \| \| \| \| **164 !** \| \| \| \| \| \| \| \| \| \| g/l \| \| \| \| \| \| \| \| \| \| (115 - 150) \| \| \| \| \| \| \| \| \| \| \| \| Hematocrit \| \| \| \| \| \| \| \| \| \| \| \| \| \| \| \| \| \| \| 49.0 \| \| \| \| \| \| \| \| \| \| % \| \| \| \| \| \| \| \| \| \| (40.0 - 51.0) \| \| \| \| \| \| \| \| \| \| \| \| Mean Corpuscular Volume \| \| \| \| \| \| \| \| \| \| \| \| \| \| \| \| \| \| \| 87 \| \| \| \| \| \| \| \| \| \| fl \| \| \| \| \| \| \| \| \| \| (82 - 98) \| \| \| \| \| \| \| \| \| \| \| \| Mean Corpuscular Hemoglobin \| \| \| \| \| \| \| \| \| \| \| \| \| \| \| \| \| \| \| 28.9 \| \| \| \| \| \| \| \| \| \| pg \| \| \| \| \| \| \| \| \| \| (27.0 - 34.0) \| \| \| \| \| \| \| \| \| \| \| \| Mean Corpuscular Hemoglobin Concentration \| \| \| \| \| \| \| \| \| \| \| \| \| \| \| \| \| \| \| 335 \| \| \| \| \| \| \| \| \| \| g/l \| \| \| \| \| \| \| \| \| \| (314 - 356) \| \| \| \| \| \| \| \| \| \| \| \| Red Cell Distribution Width \| \| \| \| \| \| \| \| \| \| \| \| \| \| \| \| \| \| \| 12.6 \| \| \| \| \| \| \| \| \| \| % \| \| \| \| \| \| \| \| \| \| (11.6 - 16.5) \| \| \| \| \| \| \| \| \| \| \| \| Platelets \| \| \| \| \| \| \| \| \| \| \| \| \| \| \| \| \| \| \| 208 \| \| \| \| \| \| \| \| \| \| 10^9^ cells/l \| \| \| \| \| \| \| \| \| \| (148 - 339) \| \| \| \| \| \| \| \| \| \| \| \| Mean Platelet Volume \| \| \| \| \| \| \| \| \| \| \| \| \| \| \| \| \| \| \| 10.8 \| \| \| \| \| \| \| \| \| \| fl \| \| \| \| \| \| \| \| \| \| (8.8 - 12.1) \| \| \| \| \| \| \| \| \| \| \| \| Plateletcrit \| \| \| \| \| \| \| \| \| \| \| \| \| \| \| \| \| \| \| 0.23 \| \| \| \| \| \| \| \| \| \| % \| \| \| \| \| \| \| \| \| \| (0.16 - 0.34) \| \| \| \| \| \| \| \| \| \| \| \| Platelet Distribution Width \| \| \| \| \| \| \| \| \| \| \| \| \| \| \| \| \| \| \| 16.1 \| \| \| \| \| \| \| \| \| \| % \| \| \| \| \| \| \| \| \| \| (9.7 - 16.7) \| \| \| \| \| \| \| \| \| \| \| \| ESR \| \| \| \| \| \| \| \| \| \| \| \| \| \| \| \| \| \| \| 5 \| \| \| \| \| \| \| \| \| \| mm/hour \| \| \| \| \| \| \| \| \| \| (2 - 15) \| \| \| \| \| \| \| \| \| \| \| | | | |
| **Blood Chemistry Analysis** | | | |
| \|  \|  \|  \|  \|  \|  \|  \|  \|  \|  \|  \|  \|  \|  \|  \|  \|  \|  \|  \|  \|  \|  \|  \|  \|  \|  \|  \|  \|  \|  \|  \|  \|  \|  \|  \|  \|  \|  \|  \|  \|  \|  \|  \|  \|  \|  \|  \|  \|  \|  \| \| --- \| --- \| --- \| --- \| --- \| --- \| --- \| --- \| --- \| --- \| --- \| --- \| --- \| --- \| --- \| --- \| --- \| --- \| --- \| --- \| --- \| --- \| --- \| --- \| --- \| --- \| --- \| --- \| --- \| --- \| --- \| --- \| --- \| --- \| --- \| --- \| --- \| --- \| --- \| --- \| --- \| --- \| --- \| --- \| --- \| --- \| --- \| --- \| --- \| --- \| \| Glucose \| \| \| \| \| \| \| \| \| \| \| \| \| \| \| \| \| \| \| 5.3 \| \| \| \| \| \| \| \| \| \| mmol/l \| \| \| \| \| \| \| \| \| \| (3.1 - 6.1) \| \| \| \| \| \| \| \| \| \| \| | | | |
| \|  \|  \|  \|  \|  \|  \|  \|  \|  \|  \|  \|  \|  \|  \|  \|  \|  \|  \|  \|  \|  \|  \|  \|  \|  \|  \|  \|  \|  \|  \|  \|  \|  \|  \|  \|  \|  \|  \|  \|  \|  \|  \|  \|  \|  \|  \|  \|  \|  \|  \| \| --- \| --- \| --- \| --- \| --- \| --- \| --- \| --- \| --- \| --- \| --- \| --- \| --- \| --- \| --- \| --- \| --- \| --- \| --- \| --- \| --- \| --- \| --- \| --- \| --- \| --- \| --- \| --- \| --- \| --- \| --- \| --- \| --- \| --- \| --- \| --- \| --- \| --- \| --- \| --- \| --- \| --- \| --- \| --- \| --- \| --- \| --- \| --- \| --- \| --- \| \| Total Protein \| \| \| \| \| \| \| \| \| \| \| \| \| \| \| \| \| \| \| 76.20 \| \| \| \| \| \| \| \| \| \| g/l \| \| \| \| \| \| \| \| \| \| (64.00 - 83.00) \| \| \| \| \| \| \| \| \| \| \| | | | |
| \|  \|  \|  \|  \|  \|  \|  \|  \|  \|  \|  \|  \|  \|  \|  \|  \|  \|  \|  \|  \|  \|  \|  \|  \|  \|  \|  \|  \|  \|  \|  \|  \|  \|  \|  \|  \|  \|  \|  \|  \|  \|  \|  \|  \|  \|  \|  \|  \|  \|  \| \| --- \| --- \| --- \| --- \| --- \| --- \| --- \| --- \| --- \| --- \| --- \| --- \| --- \| --- \| --- \| --- \| --- \| --- \| --- \| --- \| --- \| --- \| --- \| --- \| --- \| --- \| --- \| --- \| --- \| --- \| --- \| --- \| --- \| --- \| --- \| --- \| --- \| --- \| --- \| --- \| --- \| --- \| --- \| --- \| --- \| --- \| --- \| --- \| --- \| --- \| \| Total Bilirubin \| \| \| \| \| \| \| \| \| \| \| \| \| \| \| \| \| \| \| 12.5 \| \| \| \| \| \| \| \| \| \| µmol/l \| \| \| \| \| \| \| \| \| \| (3.4 - 20.5) \| \| \| \| \| \| \| \| \| \| \| | | | |
| \|  \|  \|  \|  \|  \|  \|  \|  \|  \|  \|  \|  \|  \|  \|  \|  \|  \|  \|  \|  \|  \|  \|  \|  \|  \|  \|  \|  \|  \|  \|  \|  \|  \|  \|  \|  \|  \|  \|  \|  \|  \|  \|  \|  \|  \|  \|  \|  \|  \|  \| \| --- \| --- \| --- \| --- \| --- \| --- \| --- \| --- \| --- \| --- \| --- \| --- \| --- \| --- \| --- \| --- \| --- \| --- \| --- \| --- \| --- \| --- \| --- \| --- \| --- \| --- \| --- \| --- \| --- \| --- \| --- \| --- \| --- \| --- \| --- \| --- \| --- \| --- \| --- \| --- \| --- \| --- \| --- \| --- \| --- \| --- \| --- \| --- \| --- \| --- \| \| Conjugated bilirubin (direct) \| \| \| \| \| \| \| \| \| \| \| \| \| \| \| \| \| \| \| 4.0 \| \| \| \| \| \| \| \| \| \| µmol/l \| \| \| \| \| \| \| \| \| \| (0.0 - 8.6) \| \| \| \| \| \| \| \| \| \| \| | | | |
| \|  \|  \|  \|  \|  \|  \|  \|  \|  \|  \|  \|  \|  \|  \|  \|  \|  \|  \|  \|  \|  \|  \|  \|  \|  \|  \|  \|  \|  \|  \|  \|  \|  \|  \|  \|  \|  \|  \|  \|  \|  \|  \|  \|  \|  \|  \|  \|  \|  \|  \| \| --- \| --- \| --- \| --- \| --- \| --- \| --- \| --- \| --- \| --- \| --- \| --- \| --- \| --- \| --- \| --- \| --- \| --- \| --- \| --- \| --- \| --- \| --- \| --- \| --- \| --- \| --- \| --- \| --- \| --- \| --- \| --- \| --- \| --- \| --- \| --- \| --- \| --- \| --- \| --- \| --- \| --- \| --- \| --- \| --- \| --- \| --- \| --- \| --- \| --- \| \| Creatinine \| \| \| \| \| \| \| \| \| \| \| \| \| \| \| \| \| \| \| **117.6** \| \| \| \| \| \| \| \| \| \| µmol/l \| \| \| \| \| \| \| \| \| \| (44.0 - 88.0) \| \| \| \| \| \| \| \| \| \| \| | | | |
| \|  \|  \|  \|  \|  \|  \|  \|  \|  \|  \|  \|  \|  \|  \|  \|  \|  \|  \|  \|  \|  \|  \|  \|  \|  \|  \|  \|  \|  \|  \|  \|  \|  \|  \|  \|  \|  \|  \|  \|  \|  \|  \|  \|  \|  \|  \|  \|  \|  \|  \| \| --- \| --- \| --- \| --- \| --- \| --- \| --- \| --- \| --- \| --- \| --- \| --- \| --- \| --- \| --- \| --- \| --- \| --- \| --- \| --- \| --- \| --- \| --- \| --- \| --- \| --- \| --- \| --- \| --- \| --- \| --- \| --- \| --- \| --- \| --- \| --- \| --- \| --- \| --- \| --- \| --- \| --- \| --- \| --- \| --- \| --- \| --- \| --- \| --- \| --- \| \|  \|  \|  \|  \|  \|  \|  \|  \|  \|  \|  \|  \|  \|  \|  \|  \|  \|  \|  \|  \|  \|  \|  \|  \|  \|  \|  \|  \|  \|  \|  \|  \|  \|  \|  \|  \|  \|  \|  \|  \|  \|  \|  \|  \|  \|  \|  \|  \|  \|  \| \|  \|  \|  \|  \|  \|  \|  \|  \|  \|  \|  \|  \|  \|  \|  \|  \|  \|  \|  \|  \|  \|  \|  \|  \|  \|  \|  \|  \|  \|  \|  \|  \|  \|  \|  \|  \|  \|  \|  \|  \|  \|  \|  \|  \|  \|  \|  \|  \|  \|  \| \| Urea \| \| \| \| \| \| \| \| \| \| \| \| \| \| \| \| \| \| \| **8.8** \| \| \| \| \| \| \| \| \| \| mmol/l \| \| \| \| \| \| \| \| \| \| (3.0 - 7.5) \| \| \| \| \| \| \| \| \| \| \| | | | |
| \|  \|  \|  \|  \|  \|  \|  \|  \|  \|  \|  \|  \|  \|  \|  \|  \|  \|  \|  \|  \|  \|  \|  \|  \|  \|  \|  \|  \|  \|  \|  \|  \|  \|  \|  \|  \|  \|  \|  \|  \|  \|  \|  \|  \|  \|  \|  \|  \|  \|  \| \| --- \| --- \| --- \| --- \| --- \| --- \| --- \| --- \| --- \| --- \| --- \| --- \| --- \| --- \| --- \| --- \| --- \| --- \| --- \| --- \| --- \| --- \| --- \| --- \| --- \| --- \| --- \| --- \| --- \| --- \| --- \| --- \| --- \| --- \| --- \| --- \| --- \| --- \| --- \| --- \| --- \| --- \| --- \| --- \| --- \| --- \| --- \| --- \| --- \| --- \| \| Triglycerides \| \| \| \| \| \| \| \| \| \| \| \| \| \| \| \| \| \| \| 1.3 \| \| \| \| \| \| \| \| \| \| mmol/l \| \| \| \| \| \| \| \| \| \| (0.1 - 1.7) \| \| \| \| \| \| \| \| \| \| \| | | | |
| \|  \|  \|  \|  \|  \|  \|  \|  \|  \|  \|  \|  \|  \|  \|  \|  \|  \|  \|  \|  \|  \|  \|  \|  \|  \|  \|  \|  \|  \|  \|  \|  \|  \|  \|  \|  \|  \|  \|  \|  \|  \|  \|  \|  \|  \|  \|  \|  \|  \|  \| \| --- \| --- \| --- \| --- \| --- \| --- \| --- \| --- \| --- \| --- \| --- \| --- \| --- \| --- \| --- \| --- \| --- \| --- \| --- \| --- \| --- \| --- \| --- \| --- \| --- \| --- \| --- \| --- \| --- \| --- \| --- \| --- \| --- \| --- \| --- \| --- \| --- \| --- \| --- \| --- \| --- \| --- \| --- \| --- \| --- \| --- \| --- \| --- \| --- \| --- \| \| Cholesterol \| \| \| \| \| \| \| \| \| \| \| \| \| \| \| \| \| \| \| 5.0 \| \| \| \| \| \| \| \| \| \| mmol/l \| \| \| \| \| \| \| \| \| \| (3.3 - 5.2) \| \| \| \| \| \| \| \| \| \| \| | | | |
| \|  \|  \|  \|  \|  \|  \|  \|  \|  \|  \|  \|  \|  \|  \|  \|  \|  \|  \|  \|  \|  \|  \|  \|  \|  \|  \|  \|  \|  \|  \|  \|  \|  \|  \|  \|  \|  \|  \|  \|  \|  \|  \|  \|  \|  \|  \|  \|  \|  \|  \| \| --- \| --- \| --- \| --- \| --- \| --- \| --- \| --- \| --- \| --- \| --- \| --- \| --- \| --- \| --- \| --- \| --- \| --- \| --- \| --- \| --- \| --- \| --- \| --- \| --- \| --- \| --- \| --- \| --- \| --- \| --- \| --- \| --- \| --- \| --- \| --- \| --- \| --- \| --- \| --- \| --- \| --- \| --- \| --- \| --- \| --- \| --- \| --- \| --- \| --- \| \| LDL Cholesterol \| \| \| \| \| \| \| \| \| \| \| \| \| \| \| \| \| \| \| **3.14** \| \| \| \| \| \| \| \| \| \| mmol/l \| \| \| \| \| \| \| \| \| \| (1.10 - 3.00) \| \| \| \| \| \| \| \| \| \| \| | | | |
| \|  \|  \|  \|  \|  \|  \|  \|  \|  \|  \|  \|  \|  \|  \|  \|  \|  \|  \|  \|  \|  \|  \|  \|  \|  \|  \|  \|  \|  \|  \|  \|  \|  \|  \|  \|  \|  \|  \|  \|  \|  \|  \|  \|  \|  \|  \|  \|  \|  \|  \| \| --- \| --- \| --- \| --- \| --- \| --- \| --- \| --- \| --- \| --- \| --- \| --- \| --- \| --- \| --- \| --- \| --- \| --- \| --- \| --- \| --- \| --- \| --- \| --- \| --- \| --- \| --- \| --- \| --- \| --- \| --- \| --- \| --- \| --- \| --- \| --- \| --- \| --- \| --- \| --- \| --- \| --- \| --- \| --- \| --- \| --- \| --- \| --- \| --- \| --- \| \| Aspartate Aminotransferase (AST) \| \| \| \| \| \| \| \| \| \| \| \| \| \| \| \| \| \| \| 25.2 \| \| \| \| \| \| \| \| \| \| U/l \| \| \| \| \| \| \| \| \| \| (5.0 - 34.0) \| \| \| \| \| \| \| \| \| \| \| | | | |
| \|  \|  \|  \|  \|  \|  \|  \|  \|  \|  \|  \|  \|  \|  \|  \|  \|  \|  \|  \|  \|  \|  \|  \|  \|  \|  \|  \|  \|  \|  \|  \|  \|  \|  \|  \|  \|  \|  \|  \|  \|  \|  \|  \|  \|  \|  \|  \|  \|  \|  \| \| --- \| --- \| --- \| --- \| --- \| --- \| --- \| --- \| --- \| --- \| --- \| --- \| --- \| --- \| --- \| --- \| --- \| --- \| --- \| --- \| --- \| --- \| --- \| --- \| --- \| --- \| --- \| --- \| --- \| --- \| --- \| --- \| --- \| --- \| --- \| --- \| --- \| --- \| --- \| --- \| --- \| --- \| --- \| --- \| --- \| --- \| --- \| --- \| --- \| --- \| \| Alanine Aminotransferase (ALT) \| \| \| \| \| \| \| \| \| \| \| \| \| \| \| \| \| \| \| 28.8 \| \| \| \| \| \| \| \| \| \| U/l \| \| \| \| \| \| \| \| \| \| (0.0 - 55.0) \| \| \| \| \| \| \| \| \| \| \| | | | |
| \|  \|  \|  \|  \|  \|  \|  \|  \|  \|  \|  \|  \|  \|  \|  \|  \|  \|  \|  \|  \|  \|  \|  \|  \|  \|  \|  \|  \|  \|  \|  \|  \|  \|  \|  \|  \|  \|  \|  \|  \|  \|  \|  \|  \|  \|  \|  \|  \|  \|  \| \| --- \| --- \| --- \| --- \| --- \| --- \| --- \| --- \| --- \| --- \| --- \| --- \| --- \| --- \| --- \| --- \| --- \| --- \| --- \| --- \| --- \| --- \| --- \| --- \| --- \| --- \| --- \| --- \| --- \| --- \| --- \| --- \| --- \| --- \| --- \| --- \| --- \| --- \| --- \| --- \| --- \| --- \| --- \| --- \| --- \| --- \| --- \| --- \| --- \| --- \| \| Sodium \| \| \| \| \| \| \| \| \| \| \| \| \| \| \| \| \| \| \| 138.30 \| \| \| \| \| \| \| \| \| \| mmol/l \| \| \| \| \| \| \| \| \| \| (135.00 - 145.00) \| \| \| \| \| \| \| \| \| \| \| \| Potassium \| \| \| \| \| \| \| \| \| \| \| \| \| \| \| \| \| \| \| 4.74 \| \| \| \| \| \| \| \| \| \| mmol/l \| \| \| \| \| \| \| \| \| \| (3.50 - 5.10) \| \| \| \| \| \| \| \| \| \| \| \| Chloride \| \| \| \| \| \| \| \| \| \| \| \| \| \| \| \| \| \| \| **97.0** \| \| \| \| \| \| \| \| \| \| mmol/l \| \| \| \| \| \| \| \| \| \| (98.0 - 107.0) \| \| \| \| \| \| \| \| \| \| \| | | | |
| \|  \|  \|  \|  \|  \|  \|  \|  \|  \|  \|  \|  \|  \|  \|  \|  \|  \|  \|  \|  \|  \|  \|  \|  \|  \|  \|  \|  \|  \|  \|  \|  \|  \|  \|  \|  \|  \|  \|  \|  \|  \|  \|  \|  \|  \|  \|  \|  \|  \|  \| \| --- \| --- \| --- \| --- \| --- \| --- \| --- \| --- \| --- \| --- \| --- \| --- \| --- \| --- \| --- \| --- \| --- \| --- \| --- \| --- \| --- \| --- \| --- \| --- \| --- \| --- \| --- \| --- \| --- \| --- \| --- \| --- \| --- \| --- \| --- \| --- \| --- \| --- \| --- \| --- \| --- \| --- \| --- \| --- \| --- \| --- \| --- \| --- \| --- \| --- \| \| HDL Cholesterol \| \| \| \| \| \| \| \| \| \| \| \| \| \| \| \| \| \| \| 1.30 \| \| \| \| \| \| \| \| \| \| mmol/l \| \| \| \| \| \| \| \| \| \| (0.90 - 2.60) \| \| \| \| \| \| \| \| \| \| \| | | | |
| \|  \|  \|  \|  \|  \|  \|  \|  \|  \|  \|  \|  \|  \|  \|  \|  \|  \|  \|  \|  \|  \|  \|  \|  \|  \|  \|  \|  \|  \|  \|  \|  \|  \|  \|  \|  \|  \|  \|  \|  \|  \|  \|  \|  \|  \|  \|  \|  \|  \|  \| \| --- \| --- \| --- \| --- \| --- \| --- \| --- \| --- \| --- \| --- \| --- \| --- \| --- \| --- \| --- \| --- \| --- \| --- \| --- \| --- \| --- \| --- \| --- \| --- \| --- \| --- \| --- \| --- \| --- \| --- \| --- \| --- \| --- \| --- \| --- \| --- \| --- \| --- \| --- \| --- \| --- \| --- \| --- \| --- \| --- \| --- \| --- \| --- \| --- \| --- \| \| Uric acid \| \| \| \| \| \| \| \| \| \| \| \| \| \| \| \| \| \| \| **519.4** \| \| \| \| \| \| \| \| \| \| µmol/l \| \| \| \| \| \| \| \| \| \| (202.0 - 416.0) \| \| \| \| \| \| \| \| \| \| \| | | | |
| \|  \|  \|  \|  \|  \|  \|  \|  \|  \|  \|  \|  \|  \|  \|  \|  \|  \|  \|  \|  \|  \|  \|  \|  \|  \|  \|  \|  \|  \|  \|  \|  \|  \|  \|  \|  \|  \|  \|  \|  \|  \|  \|  \|  \|  \|  \|  \|  \|  \|  \| \| --- \| --- \| --- \| --- \| --- \| --- \| --- \| --- \| --- \| --- \| --- \| --- \| --- \| --- \| --- \| --- \| --- \| --- \| --- \| --- \| --- \| --- \| --- \| --- \| --- \| --- \| --- \| --- \| --- \| --- \| --- \| --- \| --- \| --- \| --- \| --- \| --- \| --- \| --- \| --- \| --- \| --- \| --- \| --- \| --- \| --- \| --- \| --- \| --- \| --- \| \| Ionized calcium \| \| \| \| \| \| \| \| \| \| \| \| \| \| \| \| \| \| \| 1.21 \| \| \| \| \| \| \| \| \| \| mmol/l \| \| \| \| \| \| \| \| \| \| (1.03 - 1.29) \| \| \| \| \| \| \| \| \| \| \| | | | |
| \|  \|  \|  \|  \|  \|  \|  \|  \|  \|  \|  \|  \|  \|  \|  \|  \|  \|  \|  \|  \|  \|  \|  \|  \|  \|  \|  \|  \|  \|  \|  \|  \|  \|  \|  \|  \|  \|  \|  \|  \|  \|  \|  \|  \|  \|  \|  \|  \|  \|  \| \| --- \| --- \| --- \| --- \| --- \| --- \| --- \| --- \| --- \| --- \| --- \| --- \| --- \| --- \| --- \| --- \| --- \| --- \| --- \| --- \| --- \| --- \| --- \| --- \| --- \| --- \| --- \| --- \| --- \| --- \| --- \| --- \| --- \| --- \| --- \| --- \| --- \| --- \| --- \| --- \| --- \| --- \| --- \| --- \| --- \| --- \| --- \| --- \| --- \| --- \| \| Total Calcium \| \| \| \| \| \| \| \| \| \| \| \| \| \| \| \| \| \| \| **2.58** \| \| \| \| \| \| \| \| \| \| mmol/l \| \| \| \| \| \| \| \| \| \| (2.10 - 2.55) \| \| \| \| \| \| \| \| \| \| \| | | | |
| \|  \|  \|  \|  \|  \|  \|  \|  \|  \|  \|  \|  \|  \|  \|  \|  \|  \|  \|  \|  \|  \|  \|  \|  \|  \|  \|  \|  \|  \|  \|  \|  \|  \|  \|  \|  \|  \|  \|  \|  \|  \|  \|  \|  \|  \|  \|  \|  \|  \|  \| \| --- \| --- \| --- \| --- \| --- \| --- \| --- \| --- \| --- \| --- \| --- \| --- \| --- \| --- \| --- \| --- \| --- \| --- \| --- \| --- \| --- \| --- \| --- \| --- \| --- \| --- \| --- \| --- \| --- \| --- \| --- \| --- \| --- \| --- \| --- \| --- \| --- \| --- \| --- \| --- \| --- \| --- \| --- \| --- \| --- \| --- \| --- \| --- \| --- \| --- \| \| Phosphorus \| \| \| \| \| \| \| \| \| \| \| \| \| \| \| \| \| \| \| **1.91** \| \| \| \| \| \| \| \| \| \| mmol/l \| \| \| \| \| \| \| \| \| \| (1.45 - 1.78) \| \| \| \| \| \| \| \| \| \| \| \| Magnesium \| \| \| \| \| \| \| \| \| \| \| \| \| \| \| \| \| \| \| 0.79 \| \| \| \| \| \| \| \| \| \| mmol/l \| \| \| \| \| \| \| \| \| \| (0.7 - 1.05) \| \| \| \| \| \| \| \| \| \| \| \| **eGFR (Bedside Schwartz Formula)** \| \| \| \| \| \| \| \| \| \| \| \| \| \| \| \| \| \| \| 53 mL/min/1.73 m^2^ \| \| \| \| \| \| \| \| \| \|  \| \| \| \| \| \| \| \| \| \|  \| \| \| \| \| \| \| \| \| \| \| | | | |
| **Hormonal Blood Analysis** | | | |
| \|  \|  \|  \|  \|  \|  \|  \|  \|  \|  \|  \|  \|  \|  \|  \|  \|  \|  \|  \|  \|  \|  \|  \|  \|  \|  \|  \|  \|  \|  \|  \|  \|  \|  \|  \|  \|  \|  \|  \|  \|  \|  \|  \|  \|  \|  \|  \|  \|  \|  \| \| --- \| --- \| --- \| --- \| --- \| --- \| --- \| --- \| --- \| --- \| --- \| --- \| --- \| --- \| --- \| --- \| --- \| --- \| --- \| --- \| --- \| --- \| --- \| --- \| --- \| --- \| --- \| --- \| --- \| --- \| --- \| --- \| --- \| --- \| --- \| --- \| --- \| --- \| --- \| --- \| --- \| --- \| --- \| --- \| --- \| --- \| --- \| --- \| --- \| --- \| \| Free Т4 \| \| \| \| \| \| \| \| \| \| \| \| \| \| \| \| \| \| \| 13.6 \| \| \| \| \| \| \| \| \| \| pmol/l \| \| \| \| \| \| \| \| \| \| (10.1 - 17.9) \| \| \| \| \| \| \| \| \| \| \| | | | |
| \|  \|  \|  \|  \|  \|  \|  \|  \|  \|  \|  \|  \|  \|  \|  \|  \|  \|  \|  \|  \|  \|  \|  \|  \|  \|  \|  \|  \|  \|  \|  \|  \|  \|  \|  \|  \|  \|  \|  \|  \|  \|  \|  \|  \|  \|  \|  \|  \|  \|  \| \| --- \| --- \| --- \| --- \| --- \| --- \| --- \| --- \| --- \| --- \| --- \| --- \| --- \| --- \| --- \| --- \| --- \| --- \| --- \| --- \| --- \| --- \| --- \| --- \| --- \| --- \| --- \| --- \| --- \| --- \| --- \| --- \| --- \| --- \| --- \| --- \| --- \| --- \| --- \| --- \| --- \| --- \| --- \| --- \| --- \| --- \| --- \| --- \| --- \| --- \| \| Thyroid-Stimulating Hormone (TSH) \| \| \| \| \| \| \| \| \| \| \| \| \| \| \| \| \| \| \| 1.363 \| \| \| \| \| \| \| \| \| \| mU/l \| \| \| \| \| \| \| \| \| \| (0.430 - 4.200) \| \| \| \| \| \| \| \| \| \| \| | | | |
| \|  \|  \|  \|  \|  \|  \|  \|  \|  \|  \|  \|  \|  \|  \|  \|  \|  \|  \|  \|  \|  \|  \|  \|  \|  \|  \|  \|  \|  \|  \|  \|  \|  \|  \|  \|  \|  \|  \|  \|  \|  \|  \|  \|  \|  \|  \|  \|  \|  \|  \| \| --- \| --- \| --- \| --- \| --- \| --- \| --- \| --- \| --- \| --- \| --- \| --- \| --- \| --- \| --- \| --- \| --- \| --- \| --- \| --- \| --- \| --- \| --- \| --- \| --- \| --- \| --- \| --- \| --- \| --- \| --- \| --- \| --- \| --- \| --- \| --- \| --- \| --- \| --- \| --- \| --- \| --- \| --- \| --- \| --- \| --- \| --- \| --- \| --- \| --- \| \| Thyroid peroxidase antibody \| \| \| \| \| \| \| \| \| \| \| \| \| \| \| \| \| \| \| 0.0 \| \| \| \| \| \| \| \| \| \| U/ml \| \| \| \| \| \| \| \| \| \| (0.0 - 5.6) \| \| \| \| \| \| \| \| \| \| \| | | | |
| \|  \|  \|  \|  \|  \|  \|  \|  \|  \|  \|  \|  \|  \|  \|  \|  \|  \|  \|  \|  \|  \|  \|  \|  \|  \|  \|  \|  \|  \|  \|  \|  \|  \|  \|  \|  \|  \|  \|  \|  \|  \|  \|  \|  \|  \|  \|  \|  \|  \|  \| \| --- \| --- \| --- \| --- \| --- \| --- \| --- \| --- \| --- \| --- \| --- \| --- \| --- \| --- \| --- \| --- \| --- \| --- \| --- \| --- \| --- \| --- \| --- \| --- \| --- \| --- \| --- \| --- \| --- \| --- \| --- \| --- \| --- \| --- \| --- \| --- \| --- \| --- \| --- \| --- \| --- \| --- \| --- \| --- \| --- \| --- \| --- \| --- \| --- \| --- \| \| Immunoreactive insulin \| \| \| \| \| \| \| \| \| \| \| \| \| \| \| \| \| \| \| 15.9 \| \| \| \| \| \| \| \| \| \| µU/mL \| \| \| \| \| \| \| \| \| \| (2.6 - 24.9) \| \| \| \| \| \| \| \| \| \| \| | | | |
| \|  \|  \|  \|  \|  \|  \|  \|  \|  \|  \|  \|  \|  \|  \|  \|  \|  \|  \|  \|  \|  \|  \|  \|  \|  \|  \|  \|  \|  \|  \|  \|  \|  \|  \|  \|  \|  \|  \|  \|  \|  \|  \|  \|  \|  \|  \|  \|  \|  \|  \| \| --- \| --- \| --- \| --- \| --- \| --- \| --- \| --- \| --- \| --- \| --- \| --- \| --- \| --- \| --- \| --- \| --- \| --- \| --- \| --- \| --- \| --- \| --- \| --- \| --- \| --- \| --- \| --- \| --- \| --- \| --- \| --- \| --- \| --- \| --- \| --- \| --- \| --- \| --- \| --- \| --- \| --- \| --- \| --- \| --- \| --- \| --- \| --- \| --- \| --- \| \| C-peptide \| \| \| \| \| \| \| \| \| \| \| \| \| \| \| \| \| \| \| 3.41 \| \| \| \| \| \| \| \| \| \| ng/ml \| \| \| \| \| \| \| \| \| \| (1.10 - 4.40) \| \| \| \| \| \| \| \| \| \| \| | | | |
| Parathyroid Hormone (PTH) | 42.8 | pg/ml | (15 - 65) |

Oral glucose tolerance test (OGTT) with 75g of anhydrous glucose

| Time (minutes) | **0** | **30** | **60** | **90** | **120** |
| --- | --- | --- | --- | --- | --- |
| Glucose (mmol/L) | 5,30 | 9,7 | 13,8 | 13,4 | 8,40 |
| Insulin (µU/mL) | 15,90 | 34,5 | 60,4 | 87,1 | 44,7 |
| C-peptide (ng/mL) | 3,41 | 5,6 | 9,2 | 11,9 | 10,7 |
| Indices of Insulin Resistance | ISI Matsuda |  | Karo |  | HOMA |
|  | 2,73 |  | 0,33 |  | 3,75 |
| Normal range | >2,5 |  | >0,3 |  | <3,2 |

Conclusion: The results indicate impaired glucose tolerance, insulin resistance (HOMA-IR=3.75), and preserved endogenous insulin secretion.

| **General Urine Analysis** |
| --- |
|  |
| Glucose 0 mmol/l (0 - 2.8) |
| Protein  0 g/l  (0 - 0.2) |
| Bilirubin 0 µmol/l (0 - 10) |
| Urobilinogen  0 µmol/l (0 - 34) |
| Acidity (pH) 6.0 - (5 - 6) |
| Erythrocytes (hemoglobin) 0 in µl (0 - 10) |
| Ketones 0 mmol/l (0 - 0.5) |
| Nitrites Not detected |
| Leukocytes  0 in 1 µl  (0 - 25) |
| Transparency Clear Clear |
| Relative Density  1.012 g/l  (1.008-1.025) |
| Color Light yellow |

| **Abdominal Ultrasound** | |
| --- | --- |
| Liver | Location: Typical. Contours: Smooth and clear. Dimensions: Right lobe thickness: 11.8 cm; Left lobe thickness: 5.5 cm. Vascular: Portal vein diameter: 0.8 cm; Intrahepatic bile ducts and common bile duct are not dilated. Parenchyma: Structure is homogeneous. Echogenicity: Heterogeneously increased. Lesions: No space-occupying lesions identified. |
| Gallbladder | Contours: Smooth and clear. Shape: Bent/deformed. Dimensions: Length: 7.4 cm, thickness: 2.1 cm. Content: Homogeneous. Walls: Not thickened or compacted; wall thickness: 0.3 cm. Calculi/Lesions: None detected. |
| Pancreas | Contours: Smooth and clear. Dimensions (thickness): Head: 2.1 cm, Body: 1.5 cm, Tail: 2.4 cm. Structure: Moderately heterogeneous. Echogenicity: Equal to that of the liver. Pancreatic Duct (Wirsung): Not dilated. Lesions: None detected. |
| Spleen | Contours: Smooth and clear. Location: Typical. Dimensions: Length: 9.3 cm, thickness: 4.4 cm. Parenchyma: Homogeneous structure and echogenicity. Vascular: Splenic vein diameter: 0.4 cm. Lesions: None detected. |
| Conclusion | The ultrasound reveals signs consistent with early hepatic steatosis (fatty liver), gallbladder deformation, and moderate diffuse changes in the pancreas. |
| **Renal Ultrasound** | |
| Right Kidney | Contours: Smooth. Location/Mobility: Typical; no pathological mobility. Dimensions: Length: 10.2 cm, Width: 4.8 cm, Thickness: 4.4 cm. Parenchyma: Homogeneous structure and echogenicity. Thickness: 1.6 cm. Pelvicalyceal System (PCS): Not deformed or compacted. Calculi: None. Lesions: A fluid-filled lesion (cyst) up to 1.2 cm in diameter in the lower segment. |
| Left Kidney | Contours: Smooth. Location/Mobility: Typical; no pathological mobility. Dimensions: Length: 10.1 cm, Width: 4.9 cm, Thickness: 4.3 cm. Parenchyma: Homogeneous structure and echogenicity. Thickness: 1.7 cm. PCS: Not deformed or compacted. Calculi: None. Lesions: Fluid-filled lesions (cysts) up to 0.7 cm and 0.9 cm in diameter in the upper and middle segments. |
| Conclusion | Findings are consistent with cysts in both kidneys |
